# Surgical Management of a Large Dentigerous Cyst Associated With an Impacted Supernumerary Tooth: A Rare Case Report

**DOI:** 10.1002/ccr3.73015

**Published:** 2026-06-22

**Authors:** Hamid Bashiri, Maryam Mohebiniya

**Affiliations:** ^1^ Department of Oral and Maxillofacial Surgery, School of Dentistry Arak University of Medical Sciences Arak Iran; ^2^ Department of Oral and Maxillofacial Radiology, School of Dentistry Arak University of Medical Sciences Arak Iran

**Keywords:** cone‐beam computed tomography, dentigerous cyst, odontogenic cyst, supernumerary tooth

## Abstract

Dentigerous cysts are the second most common odontogenic cysts, typically forming around the crowns of impacted teeth. Their association with supernumerary teeth is rare. This report presents the successful surgical management of an unusually large dentigerous cyst linked to an impacted supernumerary tooth in the right maxilla.

## 1 Introduction

Dentigerous cysts are the most common developmental odontogenic cysts and, after radicular cysts, the second most common jaw cyst. They arise from fluid accumulation between the reduced enamel epithelium and the crown of an unerupted tooth, although several contributing factors—including follicular obstruction, increased eruptive pressure, periapical inflammation from adjacent teeth, and abnormal epithelial proliferation—have been proposed in their pathogenesis. These cysts exhibit a male predilection and are most frequently located in the mandible (approximately 70% of cases), whereas maxillary involvement is less common. Although dentigerous cysts generally present in the second and third decades of life, those associated with supernumerary teeth can occur throughout the first four decades. Radiographically, dentigerous cysts usually appear as well‐circumscribed unilocular radiolucencies, extending from the cementoenamel junction (CEJ) of the associated tooth to the CEJ of the impacted or developing tooth. The mandibular third molars are the most common site, followed by maxillary canines and maxillary third molars [1, 2, 3, 4]. Although most cases involve permanent teeth, approximately 5%–6% are associated with supernumerary teeth, particularly mesiodens [3]. Mesiodens, typically located in the anterior maxilla, may lead to a range of complications, including delayed eruption of permanent teeth, crowding, spacing issues, aberrant root development, median diastema, cyst formation, intraoral infections, tooth rotation, and root resorption. These teeth may occur as single or multiple, unilateral or bilateral, with a slight male predilection. The prognosis of dentigerous cysts is generally favorable, with low recurrence rates following complete surgical enucleation [1].

The present case is noteworthy due to the unusually large size of the cyst, its extensive superior involvement of the right maxilla, and its association with two supernumerary teeth, one of which was entirely enclosed within the cystic lumen. Such presentations are uncommon and relatively infrequently reported in the literature, warranting further documentation.

## 2 Case History/Examination

A 38‐year‐old male presented to a private oral and maxillofacial radiology center for a routine panoramic radiograph. The imaging revealed a large radiolucent lesion in the right maxilla, leading to referral to a maxillofacial surgeon for further evaluation (Figure 1). The patient's medical history was unremarkable, with no history of systemic disease, trauma, or previous maxillofacial surgery. He reported no known allergies and was not taking any medications. Regarding habits, the patient denied smoking, alcohol consumption, or parafunctional habits such as bruxism.

**FIGURE 1:**
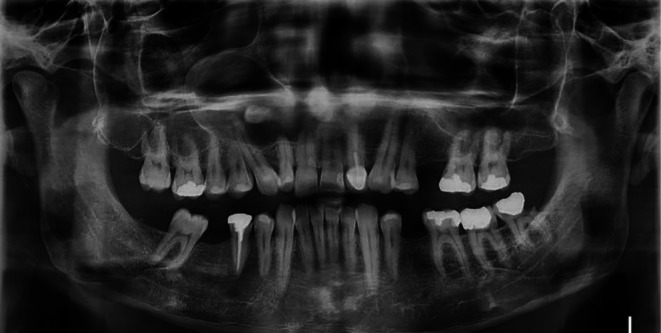
Panoramic view demonstrating a significant radiolucent lesion in the right maxilla, with an associated impacted supernumerary tooth.

The patient stated that he had been “aware of a swelling” for several months but had not sought treatment because it was painless.

On clinical examination, a slight, painless swelling of the soft tissue overlying the right maxillary sinus was observed, which the patient reported had gradually increased in size over the past year. Extraoral evaluation revealed mild facial asymmetry characterized by slight fullness in the right maxillary region, with no overlying skin changes or cervical lymphadenopathy. Sensory assessment demonstrated intact infraorbital nerve function. Additionally, intraoral examination identified a well‐defined, fluctuant swelling in the right maxillary alveolar area, with normal mucosa, no ulceration or discharge.

A thorough clinical examination prompted the surgeon to request a cone‐beam computed tomography (CBCT) scan for detailed assessment. CBCT analysis demonstrated a large, localized, unilateral lesion within the right maxillary bone (Figure 2). Multiplanar reconstructions—including axial, sagittal, coronal, and 3D views—showed that the lesion extended from the periapical region of the left central incisor to that of the right first molar, and vertically from the alveolar crest to the superior two‐thirds of the right maxillary sinus. The lesion measured 28 mm vertically, 21 mm buccopalatally, and 33.5 mm anteroposteriorly.

**FIGURE 2:**
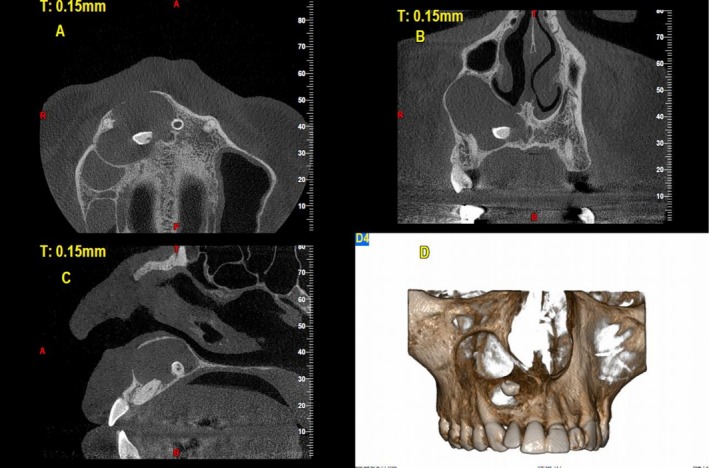
Axial (A), coronal (B), sagittal (C), and three‐dimensional (D) views clearly demonstrate the lesion's outline and extent, as well as the impacted supernumerary tooth.

Comprehensive CBCT analysis revealed the presence of two supernumerary teeth (mesiodentes). The first was located at the midline and showed no pathological association. The second was displaced laterally and was entirely encompassed within the lesion, suggesting its direct involvement in cystic development. Radiographically, the lesion appeared as a well‐circumscribed, corticated, unilocular radiolucency completely enclosing the supernumerary tooth (Figure 2).

Extensive expansion and thinning of both buccal and palatal cortical plates were noted, with bony perforation and erosion of the buccal cortical plate and anterior wall of the right maxillary sinus. The nasal floor, medial wall, and lateral anterior wall of the sinus also showed thinning. The lesion elevated the floor of the right maxillary sinus and caused superior displacement of the nasal floor. Root resorption was observed in the right central and lateral incisors, as well as the right canine, accompanied by divergence of the lateral incisor and canine. The lamina dura and periodontal ligament were not visible around the affected teeth. Mucosal thickening was present on the floor of the right maxillary sinus; however, the maxillary sinus ostium remained patent.

Based on the clinical and radiographic findings, a dentigerous cyst associated with an impacted supernumerary tooth in the anterior maxilla was considered the most likely diagnosis, pending histopathological confirmation.

## 3 Differential Diagnosis, Investigations, and Treatment

Two treatment options were considered: marsupialization and complete surgical enucleation. Marsupialization is often recommended for large cystic lesions to reduce lesion size and preserve adjacent anatomical structures; however, it requires prolonged follow‐up and may be associated with incomplete resolution or recurrence, particularly when impacted teeth are involved [5]. In the present case, the considerable size of the lesion, complete encasement of the supernumerary tooth within the cystic cavity, radiographic evidence of buccal cortical perforation, and proximity to adjacent anatomical structures favored complete surgical enucleation as the definitive treatment modality. This approach also allowed definitive histopathological evaluation.

Prior to surgery, endodontic treatment was performed on the right maxillary central incisor, lateral incisor, and canine because of radiographic evidence of root resorption, loss of lamina dura, and the anticipated surgical manipulation in close proximity to the apices of these teeth. Root canal therapy was completed 2 weeks before surgery using a conventional technique to preserve the involved teeth and minimize the risk of postoperative complications.

Following routine preoperative hematologic assessments, the patient underwent surgical cystectomy under general anesthesia. The decision to perform the procedure under general anesthesia was based on the considerable size and anterior maxillary extension of the lesion, the anticipated surgical complexity, and the need to ensure optimal patient comfort and airway control during complete enucleation. A full‐thickness mucoperiosteal sulcular incision extending from the right maxillary central incisor to the first molar, combined with a posterior vertical releasing incision, was performed to achieve adequate surgical exposure (Figure 3). After elevation of the mucoperiosteal flap, the cystic lesion was exposed and completely enucleated along with the impacted supernumerary tooth. The residual bony cavity was thoroughly curetted and irrigated with sterile saline followed by 0.2% chlorhexidine solution. No bone grafts or regenerative materials were placed, as spontaneous bone regeneration was anticipated due to the patient's age and preserved periosteal integrity.

**FIGURE 3:**
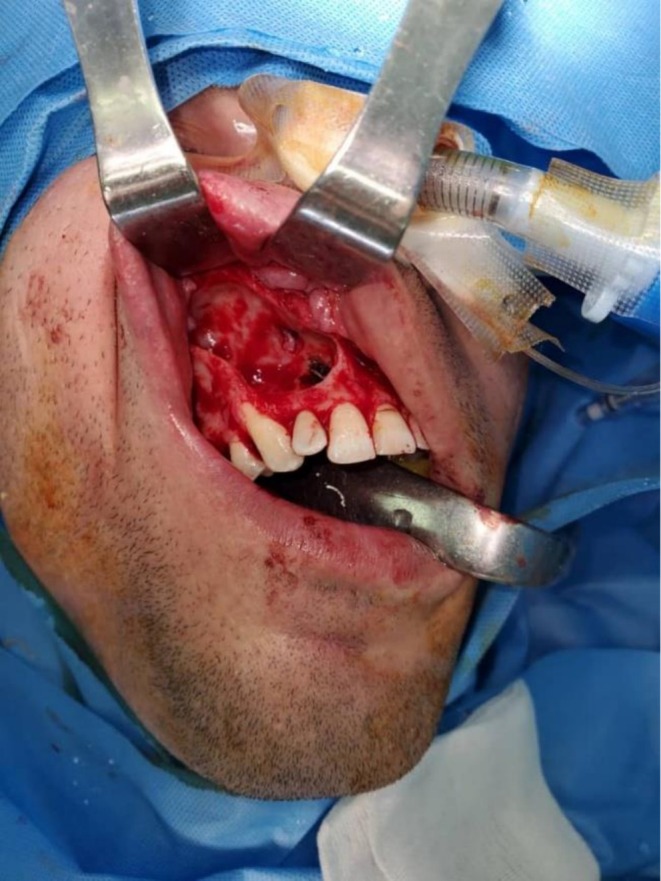
Surgical photograph illustrating the full‐thickness sulcular flap employed for cyst removal.

Intraoperatively, thinning and partial involvement of the nasal floor and the anterior wall of the maxillary sinus were observed. A small oroantral communication was identified and carefully closed using resorbable sutures to maintain separation between the oral and sinonasal cavities. The mucoperiosteal flap was repositioned and sutured with Vicryl sutures to achieve tension‐free primary closure.

Postoperative management included amoxicillin 500 mg three times daily for 7 days and analgesics as required. The patient also received detailed postoperative instructions regarding oral hygiene maintenance, avoidance of nose blowing, and prevention of mechanical trauma to the surgical site.

## 4 Conclusion and Results (Outcome and Follow‐Up)

Histopathological examination of the enucleated specimen, measuring 3 × 2 × 1 cm, revealed a cystic lesion lined by thin nonkeratinized stratified squamous epithelium resembling reduced enamel epithelium. The underlying fibrous connective tissue wall exhibited mild chronic inflammatory cell infiltration and focal vascular congestion. No evidence of epithelial dysplasia or neoplastic transformation was observed. These histopathological features were consistent with the diagnosis of a dentigerous cyst.

Postoperative recovery was uneventful. At the 1‐week and 1‐month follow‐up visits, the patient reported no complaints, and clinical examination revealed satisfactory soft‐tissue healing without evidence of infection, oroantral communication, or other complications.

At the 3‐month follow‐up, the adjacent teeth remained stable without mobility, and no signs of oronasal fistula or other postoperative complications were observed. Healing of the surgical site was progressing appropriately. A panoramic radiograph obtained at the 6‐month follow‐up demonstrated progressive bone regeneration within the surgical defect and a marked reduction in the previously observed radiolucent area. The margins of the defect appeared to gradually blend with the surrounding bone, suggesting physiologic remodeling of the maxillary structure (Figure 4). Clinical examination confirmed satisfactory soft‐tissue healing with no evidence of infection or functional impairment. Although the early postoperative outcomes were favorable, the relatively short follow‐up period of 6 months does not allow definitive conclusions regarding long‐term recurrence. Continued clinical and radiographic monitoring was therefore recommended.

**FIGURE 4:**
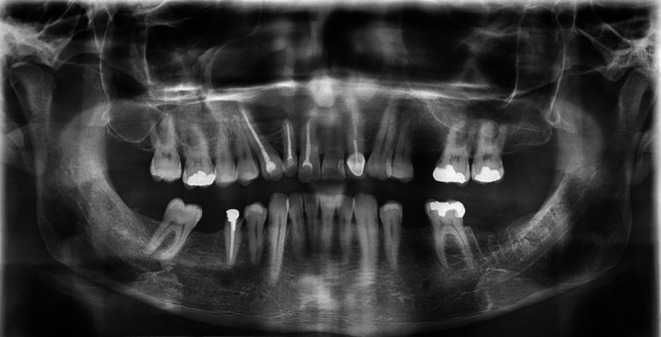
A 6‐month postoperative panoramic radiograph showed complete lesion removal and notable bone healing.

This case emphasizes the diagnostic value of correlating clinical, radiographic, and histopathological findings in cystic lesions associated with supernumerary teeth in the anterior maxilla. Complete surgical enucleation provided definitive management and resulted in favorable early clinical and radiographic outcomes. Nevertheless, given the limited follow‐up period, long‐term surveillance remains essential to confirm sustained bone regeneration and exclude recurrence.

## 5 Discussion

Dentigerous cysts are common developmental odontogenic cysts; however, large lesions associated with supernumerary teeth in the anterior maxilla are rarely reported. The present case is notable for its extensive cystic expansion, complete encasement of an impacted supernumerary tooth, and associated cortical perforation involving the nasal floor and maxillary sinus. Although typically slow‐growing and benign, dentigerous cysts may demonstrate significant local destructive potential, highlighting the importance of early diagnosis and appropriate surgical management. Diagnosis is based on combined clinical, radiographic, and histopathologic findings, typically presenting as a well‐defined pericoronal radiolucency exceeding 4 mm in diameter, lined by nonkeratinized stratified squamous epithelium and enclosing a cystic space between the enamel and surrounding tissues [1, 4, 5, 6].

In the present case, a large, unilocular pericoronal radiolucency enclosing an impacted supernumerary tooth was identified in the anterior maxilla. Given the pericoronal radiolucency associated with an impacted supernumerary tooth, a dentigerous cyst was considered the most likely provisional diagnosis. However, other odontogenic entities were systematically evaluated. Considering the location of the lesion, odontogenic keratocyst (OKC) and unicystic ameloblastoma were the primary considerations.

OKC was an important diagnostic consideration because it may occasionally present as a pericoronal radiolucency and can mimic a dentigerous cyst, especially when secondarily inflamed. However, the classic radiographic pattern of OKC—marked anteroposterior extension with minimal buccolingual expansion and scalloped margins—was not observed in this case. Furthermore, histopathologic examination did not reveal a parakeratinized epithelial lining, which allowed OKC to be confidently excluded [7].

Unicystic ameloblastoma was also included in the differential diagnosis, as it often presents as a unilocular radiolucency with sclerotic borders and may be associated with an impacted tooth [8]. Nevertheless, the enucleated specimen showed a thin, nonkeratinized epithelial lining without luminal or mural ameloblastic proliferation, and no ameloblastomatous epithelial islands were identified, thereby ruling out unicystic ameloblastoma [9].

Adenomatoid odontogenic tumor (AOT) and calcifying odontogenic cyst (COC) were considered due to their predilection for the anterior maxilla and their potential association with impacted teeth. Both lesions can appear as pericoronal radiolucencies, and in some cases, radiographic calcifications may be absent, so exclusion cannot rely solely on imaging. In the present case, however, histopathologic analysis did not demonstrate duct‐like epithelial structures suggestive of AOT or ghost cells and calcified deposits characteristic of COC, leading to exclusion of these entities [7].

Central giant cell granuloma (CGCG) was initially contemplated because of the expansile nature of the lesion. CGCG more frequently involves the mandible and usually presents as a solid, non‐cystic lesion in younger patients [10]. Intraoperative visualization of a true cystic cavity with fluid content, together with the absence of multinucleated giant cells on histology, definitively excluded CGCG.

In this case, the lesion's size, location, patient age, and exclusion of other entities supported the diagnosis of a dentigerous cyst associated with an impacted supernumerary tooth. Untreated dentigerous cysts may lead to bone deformation, permanent tooth loss, or rarely, to neoplastic transformation into squamous cell carcinoma, mural ameloblastoma, or mucoepidermoid carcinoma [11]. Treatment options include marsupialization or complete enucleation with the extraction of the associated tooth [3]. Complete enucleation not only ensures full removal of the cyst and the associated tooth but also provides an intact specimen for comprehensive histopathological evaluation, which is essential for establishing a definitive diagnosis and excluding any associated neoplastic changes.

At the 6‐month follow‐up evaluation, significant radiographic bone regeneration and clinical stability of the anterior dentition were observed, consistent with the expected healing pattern following complete enucleation of dentigerous cysts. Nevertheless, extended follow‐up is required to reliably exclude recurrence. This case underscores the importance of comprehensive diagnostic integration—encompassing radiographic assessment, intraoperative observations, and histopathologic confirmation—in accurately differentiating among clinically similar odontogenic lesions and informing appropriate surgical management.

## Author Contributions

**Hamid Bashiri:** conceptualization, investigation, supervision. **Maryam Mohebiniya:** data curation, investigation, project administration, writing – original draft, writing – review and editing.

## Funding

The authors have nothing to report.

## Consent

Written informed consent was obtained from the patient to publish this report in accordance with the journal's patient consent policy.

## Conflicts of Interest

The authors declare no conflicts of interest.

## Data Availability

Data sharing is not applicable to this article as no datasets were generated or analyzed during the current study.

## References

[1] F. Abbasi and Z. Bayat , “Dentigerous Cyst Associated With an Impacted Anterior Maxillary Supernumerary Tooth (Mesiodens): A Case Report,” Journal of Craniomaxillofacial Research 10, no. 1 (2023): 31–35.

[2] M. Mohebiniya and S. Jadidi , “Dentigerous Cyst in the Medial Wall of Maxillary Sinus,” Clinical Case Reports 12, no. 5 (2024): e8876. 38725926 10.1002/ccr3.8876 PMC11079532

[3] N. Aoki , M. Matumoto , S. Ishii , et al., “Large Dentigerous Cyst Associated With the Maxillary Impacted Supernumerary Teeth: A Rare Occurrence and Literature Review,” Journal of Dental Research, Dental Clinics, Dental Prospects 16, no. 4 (2022): 270–273. 37560494 10.34172/joddd.2022.043 PMC10407865

[4] S. Abbasi , M. Mohebiniya , S. Jadidi , and S. E. Baladeh , “Infected Dentigerous Cyst With an Unusual Finding: A Case Report,” Case Reports in Clinical Practice 10, no. 1 (2025): 12–16.

[5] P. Batra , S. G. Batra , D. Ahuja , and R. Batra , “Management and Rehabilitation of Dentigerous Cyst With 10‐Year Follow‐Up: A Case Report,” Cureus 16, no. 8 (2024): e67867. 39328611 10.7759/cureus.67867 PMC11426947

[6] H. Kaur , S. Mahajan , P. Marya , R. Goyal , and P. H. Sharma , “Management of a Dentigerous Cyst Associated With Mesiodens: A Case Report,” Dentistry: Advanced Research 4, no. 4 (2017): 139.

[7] S. Mallya and E. Lam , White and Pharoah's Oral Radiology: Principles and Interpretation (Elsevier Health Sciences, 2018).

[8] A. M. Rajasekaran , C. Prasad , J. Balaji , K. Arunkumar , and P. Raghavan , “Unicystic Ameloblastoma—A Case Series,” Indian Journal of Dental Research 34, no. 1 (2023): 104–107. 37417069 10.4103/ijdr.ijdr_521_22

[9] J. Peter , T. B. Emmatty , B. Jose , et al., “Unicystic Ameloblastoma Associated With Primary Mandibular Second Molar: A Case Report,” International Journal of Clinical Pediatric Dentistry 16 (2023): 335–338. 38268624 10.5005/jp-journals-10005-2701 PMC10804306

[10] M. Tahmasbi‐Arashlow , P. B. Patel , M. K. Nair , H. Liang , and Y. S. L. Cheng , “Cone‐Beam Computed Tomographic Imaging of Central Giant Cell Granuloma: A Comprehensive Review,” Imaging Science in Dentistry 52, no. 2 (2022): 123–131. 35799967 10.5624/isd.20220130 PMC9226240

[11] P. Nanwani , “Maxillary Dentigerous Cyst Associated With Supernumerary Tooth,” Bengal Journal of Otolaryngology and Head Neck Surgery 27, no. 1 (2019): 27.

